# Why Provide an Opinions Section in *PLoS Pathogens*? 

**DOI:** 10.1371/journal.ppat.0010013

**Published:** 2005-09-30

**Authors:** Marianne Manchester

“There were never in the world two opinions alike, any more than two hairs or two grains. Their most universal quality is diversity.”—Michel de Montaigne, French essayist

The global scope of pathogen research—a dynamic field that spans basic science, public health, epidemiology, vaccines, infectious diseases, genetics, and population dynamics—ensures that a great diversity of opinions and perspectives exist within its community of scientists. The verbal expression of such points of view, shared expertise, and opinion for its own sake may be readily witnessed at any scientific or faculty meeting. But in research articles as well as in reviews, it seems scientists are increasingly discouraged from broad speculation or from making statements that border on the provocative. This trend risks dampening the rigor and enthusiasm found in the vibrant pathogen research field.

Thus, it was the opinion of the editorial board of this new open-access journal that it was important to provide an Opinions forum. We recognize that there are important questions we can raise, and points of view to put forth—especially at a time when the field is advancing in many and key directions. The Opinions section of *PLoS Pathogens* is intended to provide a place for pathogen researchers to express their views on topics ranging from issues in experimental science to those involving science and public-health policy, education and training, and funding. And it is also a forum in which colleagues can bring another view in response to a stated opinion or observation, thus enriching the dialogue.

Our view for the Opinions section is to include original, editorial-length pieces that convey a concise and well-stated point of view on a timely subject. We will encourage authors to rely heavily on their unique perspective as scientists in the field, and will strive to allow plenty of leeway for speculation and model building, more than for a regular research article or a review article. An important goal of Opinions is to reach the collective *PLoS Pathogens* community; therefore, it will be important that each piece provides specific examples supporting the points made that relate to pathogenesis in the broader sense, rather than focusing exclusively on a single pathogen.

Opinions published in *PLoS Pathogens* can have an immediate and powerful effect on your colleagues in the pathogen research community and in the broader community of scientists. The open-access format of *PLoS Pathogens* ensures that Opinions are immediately accessible to the widest possible global audience to read, digest, and respond to.

While Opinion articles are typically solicited directly from potential authors by the Opinions editor and the editorial board, suggestions for topics of interest and import are welcomed, and should be directed to the Opinions editor, Marianne Manchester (marim@scripps.edu).

##

**About Opinion Articles**

- Published monthly online
- Invited and submitted articles are considered
- Target length of 1,000 words
- Subject to limited peer review
- Addressing topics of interest to the wider pathogen research community

